# Clinical Characteristics and Surgical Outcomes of Pediatric Ovarian Torsion: A 15-Year Single-Center Experience

**DOI:** 10.3390/children13081118

**Published:** 2026-08-21

**Authors:** Ivana Fratrić, Radoica Jokić, Jelena Antić, Stanislava Bodonji, Miloš Pajić

**Affiliations:** 1 Faculty of Medicine, University of Novi Sad, 21000 Novi Sad, Serbia; radoica.jokic@mf.uns.ac.rs (R.J.); jelena.antic@mf.uns.ac.rs (J.A.); milos.pajic@mf.uns.ac.rs (M.P.); 2 Institute for Children and Youth Healthcare of Vojvodina, 21000 Novi Sad, Serbia; stanislava.bodonji@izzzdiovns.rs

**Keywords:** ovarian torsion, pediatric surgery, ovarian preservation, oophorectomy, detorsion, oophoropexy, pediatric gynecology

## Abstract

**Highlights:**

**What are the main findings?**

- Ovarian preservation was achieved in 62.5% of patients overall and 72.9% of patients with postnatal ovarian torsion.
- Prenatal ovarian torsion represented a clinically distinct subgroup with a substantially higher oophorectomy rate than postnatal torsion (100% vs. 27.1%).

**What are the implications of the main findings?**

- The findings support ovarian-sparing management in pediatric ovarian torsion whenever technically feasible, while recognizing prenatal torsion as a distinct clinical entity.
- Preoperative gynecologist consultation was associated with a numerically lower unadjusted oophorectomy rate, but this association was not statistically significant after adjustment and should not be interpreted as causal.

**Abstract:**

Background: Ovarian torsion is a pediatric surgical emergency in which timely diagnosis and preservation of ovarian tissue are important considerations for future reproductive and endocrine function. This study aimed to describe the clinical characteristics, diagnostic evaluation, surgical management, and outcomes of pediatric ovarian torsion and explore factors associated with ovarian preservation and oophorectomy. Methods: A retrospective, single-center cohort study was conducted including 56 girls with surgically confirmed ovarian torsion treated at a tertiary pediatric center between January 2010 and December 2025. Secondary analyses of factors associated with oophorectomy were considered exploratory. Demographic, clinical, imaging, operative, and outcome data were extracted from medical records. Variables included symptom duration, degree of torsion, laterality, diagnostic assessment, gynecologist consultation, associated pathology, intraoperative macroscopic color change following detorsion, oophoropexy, oophorectomy, postoperative complications, and recurrence. Univariable comparisons were performed using the Mann–Whitney U test, chi-square test, Fisher’s exact test, or Monte Carlo exact test, as appropriate. Exploratory multivariable logistic regression, supplemented by Firth penalized logistic regression, was performed in the postnatal cohort to assess factors associated with oophorectomy. Results: The median age at diagnosis was 10 years (range 0–17) and the median duration of symptoms before admission was 18 h (IQR 8–72). Ovarian preservation was achieved in 35 patients (62.5%), whereas complete oophorectomy or adnexectomy was performed in 21 (37.5%). Complete macroscopic color change following detorsion was observed in 19 patients (33.9%), partial color change in 13 (23.2%), and no macroscopic color change in 24 (42.9%). Prenatal ovarian torsion represented a clinically distinct subgroup and was associated with a high oophorectomy rate (8/8, 100%), compared with 13/48 (27.1%) among patients with postnatal torsion (*p* < 0.001). In the postnatal cohort, complete oophorectomy or adnexectomy occurred in 19.4% of patients with preoperative gynecologist consultation and 41.2% of those without consultation (unadjusted OR 0.34, 95% CI 0.09–1.28; *p* = 0.173). In exploratory multivariable analysis adjusting for age and calendar period, preoperative gynecologist consultation was not independently associated with oophorectomy (adjusted OR 0.41, 95% CI 0.09–1.78; *p* = 0.234); Firth penalized regression yielded similar results. Associated pathology was identified in 66.1% of patients. Documented recurrence occurred in three patients (5.4%), and postoperative complications were uncommon (3.6%). Conclusions: Ovarian preservation was achieved in more than half of pediatric patients with ovarian torsion, whereas prenatal torsion was associated with a high oophorectomy rate. Preoperative gynecologist consultation was associated with a numerically lower unadjusted oophorectomy rate; however, this association was not statistically significant in either unadjusted or adjusted analyses, and residual confounding and changes in clinical practice over the 15-year study period cannot be excluded.

## 1. Introduction

Ovarian torsion (OT) is a gynecological surgical emergency resulting from rotation of the ovary around its vascular pedicle and supporting structures, with possible involvement of the fallopian tube [1]. Progressive vascular compromise may lead to ovarian ischemia, tissue necrosis, and loss of ovarian function, making timely recognition and preservation of ovarian tissue particularly important in children and adolescents.

Diagnosis of pediatric ovarian torsion remains challenging because clinical presentation and laboratory findings are often nonspecific. Ultrasonography with Doppler evaluation is the first-line imaging modality, although preserved arterial or venous flow does not exclude torsion because of the dual ovarian blood supply and the possibility of incomplete or intermittent torsion. Consequently, diagnosis may be delayed, potentially affecting ovarian viability and subsequent surgical management.

Surgical management has shifted from oophorectomy based largely on ischemic or necrotic intraoperative appearance toward detorsion and ovarian preservation. Gross ovarian appearance does not reliably predict viability, and functional recovery has been reported following detorsion even in severely ischemic-appearing ovaries [2,3,4]. Current recommendations therefore favor detorsion and ovarian preservation whenever technically feasible rather than oophorectomy based solely on macroscopic appearance [5,6]. Importantly, ovarian preservation at surgery and immediate macroscopic improvement following detorsion represent intraoperative findings and should be distinguished from postoperative anatomical viability and long-term ovarian function.

Despite this shift toward ovarian-sparing management, factors associated with ovarian preservation versus oophorectomy in pediatric patients remain incompletely defined, and available pediatric studies have reported heterogeneous findings. Surgical decision-making may be influenced by patient age, clinical presentation, diagnostic pathway, associated pathology, intraoperative findings, and specialist involvement. Gynecologist consultation was therefore considered a clinically relevant factor because specialist involvement may influence diagnostic assessment and surgical decision-making regarding ovarian preservation. In addition, prenatal ovarian torsion represents a clinically distinct presentation, with differences in timing, diagnostic pathway, ovarian viability, and management that may substantially influence overall surgical outcomes when analyzed together with acute postnatal torsion. Unlike large multicenter and administrative-database studies, a detailed institutional series allows patient-level evaluation of the diagnostic pathway, specialist involvement, associated pathology, and intraoperative findings that are often incompletely captured in registry-based data. Therefore, the primary objective of this 15-year single-center retrospective study was to describe the clinical characteristics, diagnostic evaluation, surgical management, and outcomes of pediatric ovarian torsion. We hypothesized that prenatal presentation, as a clinically distinct subgroup, would be associated with a higher likelihood of oophorectomy. Secondary analyses explored other clinical, diagnostic, and operative factors associated with ovarian preservation or oophorectomy.

## 2. Materials and Methods

### 2.1. Study Design and Setting

This study was designed as a retrospective cohort study including pediatric patients diagnosed with ovarian torsion and treated at the Institute for Children and Youth Healthcare of Vojvodina, a tertiary academic referral center, specifically at the Clinic for Pediatric Surgery in Novi Sad, Serbia, between January 2010 and December 2025.

### 2.2. Study Population and Eligibility Criteria

**Figure 1:**
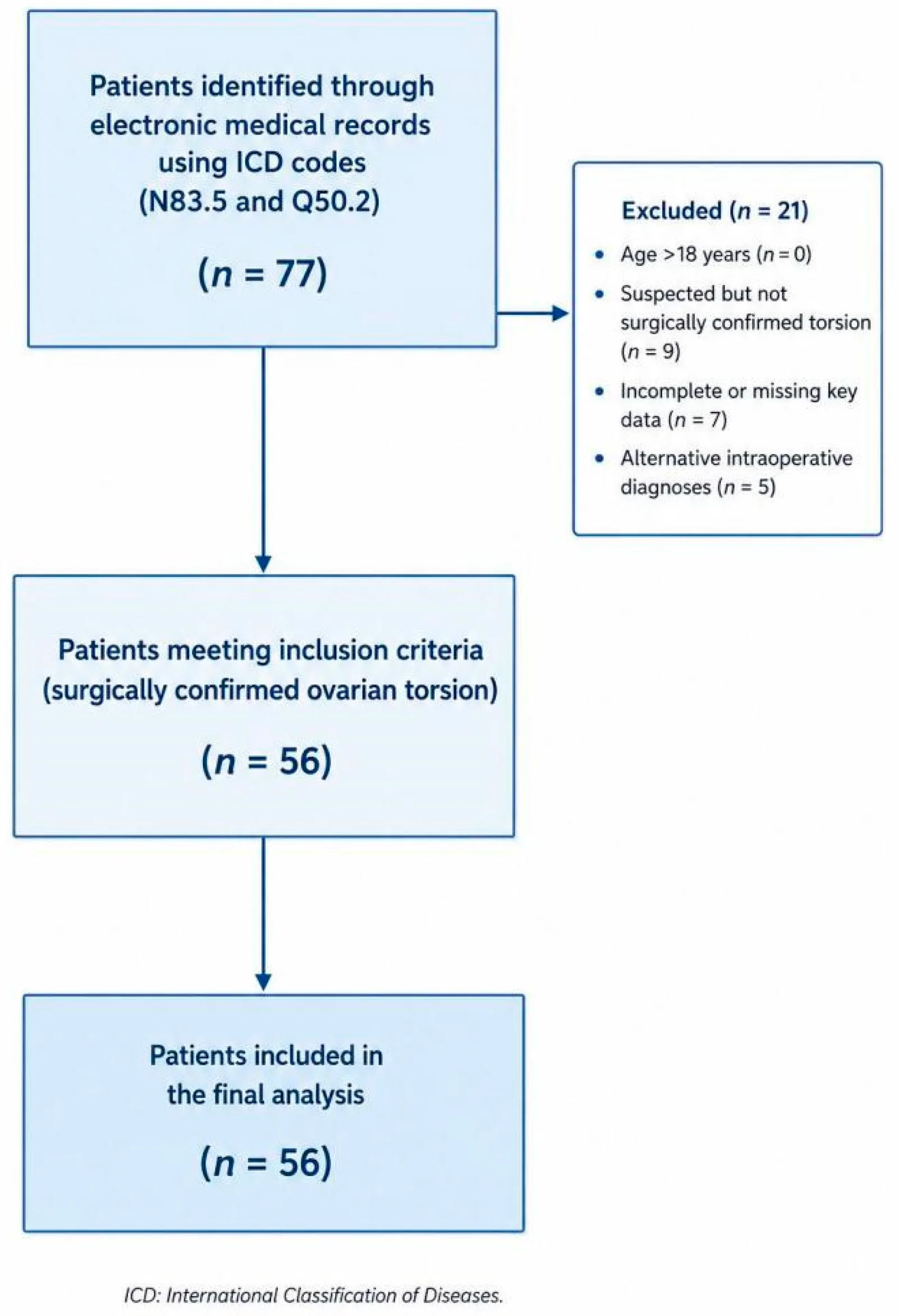
Flow diagram of patient selection and study inclusion.

Potentially eligible cases were identified through institutional electronic medical records using the predefined International Classification of Diseases (ICD) codes N83.5 (torsion of the ovary, ovarian pedicle, and fallopian tube; Torsio ovarii, pedunculi ovarii et tubae uterinae) and Q50.2 (congenital torsion of the ovary; Torsio ovarii congenita). A total of 77 patients were initially identified (67 with code N83.5 and 10 with code Q50.2). Medical and operative records were subsequently reviewed for all identified patients to verify eligibility and confirm surgically documented ovarian or adnexal torsion. Available histopathological findings were also reviewed where applicable. Of the 77 initially identified patients, 21 were excluded: nine had suspected ovarian torsion without surgical confirmation, seven had insufficient medical documentation to confirm eligibility or the primary study outcomes, and five had an alternative intraoperative diagnosis inconsistent with ovarian or adnexal torsion. Consequently, 56 patients with surgically confirmed ovarian or adnexal torsion and sufficient documentation were included in the final analysis (Figure 1).

Inclusion criteria were: (1) female patients aged 0–18 years; (2) surgically confirmed diagnosis of ovarian or adnexal torsion; and (3) availability of sufficient medical documentation to confirm eligibility and primary study outcomes. Exclusion criteria were: (1) age >18 years; (2) suspected but not surgically confirmed torsion; (3) incomplete or missing key clinical or operative data; and (4) alternative intraoperative diagnoses not consistent with torsion.

Specific clinical presentations were handled as follows. One patient had bilateral ovarian torsion, with oophorectomy performed on one side and preservation of the contralateral ovary; for patient-level outcome analyses, this patient was classified in the oophorectomy group because an oophorectomy had been performed. One patient had adnexal torsion involving both the ovary and fallopian tube; following detorsion, the ovary showed macroscopic improvement and was preserved, whereas the fallopian tube remained nonviable with features of hematosalpinx, and salpingectomy was performed. No isolated tubal torsion was identified. Three patients had associated paratubal cysts, which were excised during the same surgical procedure. Recurrent presentation was defined as a new, clinically distinct episode of ovarian torsion occurring after the initial surgically treated episode and subsequently confirmed at repeat surgery. Recurrent episodes were recorded as outcomes of the index cases and were not counted as additional patients in the study cohort.

### 2.3. Data Collection and Variables

Prior to data extraction, the study protocol, including the study objectives, eligibility criteria, variables of interest, primary outcome, and main statistical analysis plan, was developed and agreed upon by the investigators. Thus, the principal variables and planned analyses were defined before review and extraction of individual patient data. Additional analyses performed to further characterize observed associations were considered exploratory. Data were extracted from medical records using a standardized data collection form by a single trained researcher to ensure consistency. Data extraction was independently verified by a second investigator for accuracy. The following variables were collected: duration of symptoms prior to admission (in hours), obtained from history charts based on patient or caregiver reports; degree of torsion, assessed intraoperatively from surgical reports (defined as the number of rotations, with one rotation corresponding to 360°); laterality (left or right ovary); preoperative diagnostic evaluation, including whether ultrasonography was performed and whether ovarian torsion was suspected primarily on clinical grounds; involvement of a gynecologist in patient management; performance of oophoropexy; type of surgical procedure (ovarian preservation vs. oophorectomy); postoperative complications; and recurrence of torsion. Clinical presentation variables additionally extracted from the medical records included the presence of abdominal or pelvic pain, vomiting, fever, and a palpable abdominal or pelvic mass. Preoperative laboratory data included the leukocyte count and C-reactive protein (CRP) concentration when available. Because laboratory testing was not uniformly performed throughout the 15-year study period, missing values were reported explicitly and no imputation was performed. For patient-level outcome classification, ovarian preservation was defined as retention of any ovarian tissue without complete oophorectomy or adnexectomy and therefore included conservative procedures involving partial ovarian or associated lesion resection when ovarian tissue was retained. Time from hospital admission to surgery was defined as the interval from documented hospital admission to the start of the operative procedure in which ovarian or adnexal torsion was confirmed. In clinical practice, once the diagnosis of ovarian torsion was established—either on clinical grounds or by ultrasonography—the decision for urgent surgery was made without intentional delay. Therefore, this interval closely reflected the diagnostic-to-operative pathway, although exact times of ultrasound confirmation and surgical decision were not available for all patients. Preoperative ultrasonography was performed when clinically indicated and available. All ultrasonographic examinations included in the present analysis were performed at our institution. In patients who underwent gynecologic consultation, an additional ultrasonographic examination could also be performed by the consulting gynecologist as part of the clinical assessment. For the present retrospective study, written ultrasonography reports were reviewed; original stored ultrasound images were not systematically re-evaluated. Ultrasonographic findings were not necessarily diagnostic or clearly suggestive of ovarian/adnexal torsion, and the decision to proceed to surgery was therefore based on the overall clinical presentation and persistence of symptoms when clinical suspicion of torsion remained. Available preoperative ultrasonography reports were additionally reviewed for ovarian enlargement and dimensions/volume, peripheral follicles, stromal edema, free pelvic fluid, whirlpool sign, Doppler flow characteristics, and associated ovarian or adnexal masses. Because ultrasonographic reporting was not standardized throughout the 15-year study period, findings not explicitly described in the original report were classified as “not reported” rather than “absent.” Only clearly documented variables were included in the analysis. Associated pathology was defined as a documented ovarian, adnexal, or other relevant associated lesion identified in relation to the torsion presentation and classified using a combination of preoperative ultrasonographic findings, intraoperative findings, and histopathological examination when available. Operative findings were used to identify lesions directly visualized during surgery, while histopathological findings were used to further characterize excised specimens when pathological examination was performed. Histopathological confirmation was therefore not required for classification of associated pathology in every case. For surgically excised lesions with available histopathological examination, lesion classification was based on the terminology documented in the final histopathology report. The terms “dermoid cyst” and “teratoma” therefore reflect the original pathological diagnoses and were retained as separate categories. All data were anonymized prior to analysis. Exact timestamps for ultrasonography, the surgical decision, skin incision, and detorsion were not systematically recorded in the available medical documentation; therefore, admission-to-ultrasound, ultrasound-to-surgical decision, decision-to-incision, and admission-to-detorsion intervals could not be reliably calculated.

Gynecologist consultation was defined as documented preoperative involvement of a gynecologist in the diagnostic or management pathway. This included in-person gynecologic assessment, telephone consultation, and, in selected cases, gynecologic assessment with subsequent attendance or participation in the operating theatre. Postoperative gynecologic consultation alone was not classified as preoperative gynecologist consultation. The type and timing of gynecologic involvement were retrospectively extracted from the available medical records. For comparison of patients with and without preoperative gynecologist consultation, baseline and clinical characteristics included age, calendar period, symptom duration, degree of torsion, associated pathology, suspected ovarian/adnexal mass on preoperative ultrasonography, and operative approach. Surgeon specialty was not analyzed because the primary operating surgeon and the extent of joint pediatric surgical and gynecologic participation could not be consistently classified from the retrospective records.

Prenatal ovarian torsion was considered a clinically distinct subgroup because the timing of torsion, symptom onset, diagnostic pathway, and ovarian viability differ substantially from those of acute postnatal torsion. Eight patients were retrospectively classified as having ovarian torsion that had occurred prenatally. As antenatal imaging documentation was not systematically available, this classification was based primarily on intraoperative findings considered consistent with longstanding torsion originating before birth, rather than on a documented antenatal diagnosis. Prenatal cases were described separately and excluded from analyses of symptom duration, admission-to-surgery interval, degree of torsion in relation to surgical outcome, gynecologist consultation, and factors associated with oophorectomy. These analyses were therefore performed in the postnatal cohort only (*n* = 48).

For age-group analyses, patients aged 0–12 years were classified as preadolescent and those aged 13–18 years as adolescent. Menarchal and pubertal status could not be reliably assessed because these data were not consistently documented in the available medical records; therefore, analyses were based on predefined age categories. When the medical record described a prolonged history of intermittent or recurrent abdominal pain without clearly documenting the onset of the acute symptomatic episode leading to hospital admission, symptom duration was considered unavailable and treated as missing for analyses involving this variable. This approach was used to avoid interpreting prolonged histories of intermittent or recurrent pain as continuous symptom duration attributable to an acute torsion episode.

### 2.4. Surgical Management and Intraoperative Assessment

Surgical management was determined according to intraoperative findings and the clinical circumstances at the time of surgery. Operative approach was classified as laparoscopy, laparotomy, or conversion from laparoscopy to laparotomy. Operative procedures were retrospectively extracted from the surgical reports and included detorsion, excision or resection of associated ovarian or paratubal lesions; cyst drainage or decompression by puncture, fenestration, or unroofing; oophoropexy; salpingectomy; oophorectomy; and salpingo-oophorectomy (adnexectomy), as applicable. Additional procedures performed during the same operation, including appendectomy and hernia repair, were also recorded. Because more than one procedure could be performed during the same operation, individual procedural categories were not mutually exclusive. Following detorsion, the ovary was observed intraoperatively for approximately 20–30 min before macroscopic color change was assessed. Irrigation was routinely performed during the observation period, and cyst decompression was performed when an associated ovarian cyst was present. Macroscopic color change after detorsion was retrospectively classified as complete, partial, or absent based on detailed descriptions in the operative reports. Complete macroscopic color change was defined as restoration toward a normal pink appearance, partial change as visible improvement in color without complete normalization, and absent change as no documented improvement in ovarian color during the intraoperative observation period. These categories were retrospectively applied during data extraction and were not based on an objective assessment of ovarian perfusion.

For patients undergoing oophorectomy, the documented intraoperative indication for ovarian removal was retrospectively extracted from the operative report. Reasons were categorized according to the operative description, including autoamputation, severe tissue disintegration precluding reconstruction, uncontrolled bleeding, suspected malignancy, organized or calcified necrotic tissue in prenatal torsion, or other explicitly documented reasons. When no specific reason beyond perceived nonviability was documented, this was recorded separately rather than inferred retrospectively.

### 2.5. Follow-Up

Postoperative follow-up was performed according to the same institutional surveillance approach irrespective of surgical treatment. Follow-up consisted of clinical assessment at scheduled outpatient visits, with additional investigations performed when clinically indicated. Patients were also advised to undergo gynecologic follow-up; however, subsequent gynecologic assessments were not systematically available in our institutional records and were therefore not included in the present analysis. Follow-up duration was calculated from the date of surgery to the last documented clinical assessment available in our institutional records. This calculation was restricted to patients with at least one documented postoperative outpatient visit. Recurrence was identified from documented subsequent presentations and treatment within our institution. A history of ovarian torsion preceding the index admission, including episodes diagnosed or treated at another institution, was not classified as recurrence for the purposes of this study. Because follow-up outside our institution was not systematically available, recurrent torsion diagnosed or treated elsewhere could not be captured. Postoperative complications were retrospectively classified according to the Clavien–Dindo classification based on the treatment required. Recurrent ovarian torsion was considered a separate follow-up outcome and was not classified as a postoperative complication. Subsequent operations performed for recurrent torsion were recorded separately from reoperations related to postoperative complications. Other postoperative outcomes assessed included readmission, reoperation, infection, thromboembolic events, and mortality.

### 2.6. Statistical Analysis

Diagnostic misclassification was minimized by restricting the analysis to surgically confirmed cases. Data extraction was standardized and independently verified by a second investigator.

Continuous variables were assessed for distribution using the Shapiro–Wilk test. Normally distributed variables were summarized as mean ± standard deviation, whereas non-normally distributed or highly skewed variables were summarized as median and interquartile range (IQR). For between-group comparisons using the Mann–Whitney U test, continuous variables were reported as median and interquartile range (IQR), together with the Mann–Whitney U statistic and *p*-value. Categorical variables were presented as absolute numbers and percentages. Differences between groups were analyzed using the chi-square (χ^2^) test for categorical variables and the Mann–Whitney U test for continuous variables. Follow-up duration was compared between patients undergoing complete oophorectomy/adnexectomy and those with ovarian preservation using the Mann–Whitney U test; this analysis was restricted to patients with at least one documented postoperative outpatient visit. For categorical variables with small expected cell counts, Fisher’s exact test or an exact test based on Monte Carlo simulation (1000 samples) was used, as appropriate. Patients with missing values for a given variable were excluded only from analyses involving that variable (complete-case analysis). A *p*-value < 0.05 was considered statistically significant. Statistical analyses were performed using SPSS version 25.0. Analyses of symptom duration, admission-to-surgery interval, degree of torsion in relation to surgical outcome, gynecologist consultation, and factors associated with oophorectomy were restricted to the postnatal cohort (*n* = 48), as prespecified above.

Given the limited number of oophorectomy events, multivariable analyses were considered exploratory and deliberately restricted to a small number of clinically justified covariates. In the postnatal cohort, conventional logistic regression was performed with oophorectomy as the dependent variable and age, preoperative gynecologist consultation, and calendar period (2010–2017 vs. 2018–2025) as covariates. Because of the limited number of outcome events and the potential for small-sample bias, Firth penalized logistic regression using the same covariates was performed as a robustness analysis. Adjusted odds ratios (ORs) with 95% confidence intervals (CIs) were reported. No automated or univariable *p*-value-based variable selection was performed.

To explore temporal changes in clinical practice, the postnatal cohort was additionally divided into two calendar periods (2010–2017 and 2018–2025). Rates of oophorectomy, laparoscopic approach, gynecologist consultation, documented preoperative ultrasonography, and oophoropexy were compared between the two periods. These calendar-period comparisons were considered exploratory.

The primary outcome was oophorectomy versus ovarian preservation. The principal analyses focused on clinically prespecified factors potentially associated with this outcome. Given the number of additional comparisons performed and the limited sample size, all secondary and post hoc analyses were considered exploratory. No formal adjustment for multiple comparisons was applied; therefore, *p*-values from these exploratory analyses should be interpreted cautiously and in conjunction with effect estimates and 95% confidence intervals rather than as confirmatory evidence.

As a sensitivity analysis, the association between symptom duration and oophorectomy was re-evaluated after excluding extreme symptom-duration values of ≥168 h (7 days).

### 2.7. Ethical Considerations

The study was approved by the Ethics Committee of the Institute for Children and Youth Healthcare of Vojvodina (Approval No.: 17/74-2, Date: 30 December 2025). The study was conducted in accordance with the Declaration of Helsinki and institutional ethical standards. Written consent for the use of medical-record data for scientific and research purposes had been routinely obtained from the parents or legal guardians at the time of hospital admission and was available for all patients included in the study. No additional study-specific informed consent was obtained because the present retrospective analysis used previously collected and anonymized medical-record data.

The study was reported in accordance with the Strengthening the Reporting of Observational Studies in Epidemiology (STROBE) Statement.

## 3. Results

### 3.1. Patient Characteristics

A total of 56 pediatric patients with surgically confirmed ovarian torsion were included in the final analysis. The median age at diagnosis was 10 years (range, 0–17 years); 39 patients (69.6%) were preadolescent and 17 (30.4%) were adolescents. Eight patients (14.3%) were retrospectively classified as having ovarian torsion that had occurred prenatally.

Among patients with postnatal torsion, the median duration of symptoms before hospital admission was 18 h (IQR, 8–72 h), and the median admission-to-surgery interval was 20.3 h (IQR, 4.5–28.7 h) among the 45 patients with available data. Abdominal or pelvic pain was the predominant presenting symptom (47/48, 97.9%), followed by vomiting (24/48, 50.0%).

Baseline clinical, diagnostic, and operative characteristics are summarized in Table 1.

### 3.2. Ovarian Torsion Retrospectively Classified as Prenatal

Eight patients were retrospectively classified as having ovarian torsion that had occurred prenatally and underwent surgery during the neonatal period (6–14 days of age). Prenatal timing was primarily inferred from operative findings consistent with longstanding torsion because antenatal imaging documentation was not systematically available. In one patient, an intrauterine ovarian cyst had been documented.

All eight patients had advanced ovarian or adnexal nonviability at surgery and underwent complete oophorectomy or adnexectomy. Detailed case-level characteristics are provided in Supplementary Table S1.

Given the clinically distinct presentation of prenatal torsion, these patients were analyzed separately and excluded from subsequent analyses dependent on acute symptom onset, diagnostic pathway, gynecologist consultation, and factors associated with oophorectomy.

### 3.3. Postnatal Ovarian Torsion

Presenting clinical findings did not differ significantly between patients undergoing complete oophorectomy and those with ovarian preservation. Leukocyte counts were available in 42/48 patients (87.5%) and were similar between groups (median 12.1 [IQR 10.4–15.7] vs. 11.5 [IQR 8.1–14.6] ×10^9^/L; *p* = 0.152). CRP measurements were available in 27/48 patients (56.3%) and were numerically higher in the oophorectomy group (median 36.3 [IQR 3.3–71.4] vs. 0.76 [IQR 0.45–3.46] mg/L), although the difference was not statistically significant (*p* = 0.056).

Documented preoperative ultrasonography was available in 42/48 patients (87.5%). Ultrasonographic reporting was heterogeneous, with suspected ovarian/adnexal masses or prominent cystic/mass-like lesions documented in 21/42 patients (50.0%) and Doppler assessment specifically reported in 23/42 (54.8%). Detailed ultrasonographic findings are provided in Supplementary Table S2.

Preoperative gynecologist consultation was documented in 31/48 patients (64.6%). Patients who underwent consultation were older than those without consultation (median 13.0 [IQR 9.5–14.5] vs. 7.0 [IQR 2.0–12.0] years; *p* = 0.004). A suspected ovarian/adnexal mass on preoperative ultrasonography was numerically more frequent among patients with consultation (54.8% vs. 23.5%; *p* = 0.067), whereas symptom duration, degree of torsion, associated pathology, calendar period, and laparoscopic approach did not differ significantly between groups (Table 2).

Complete oophorectomy or adnexectomy was performed in 6/31 patients (19.4%) with preoperative gynecologist consultation and 7/17 (41.2%) without consultation. This difference was not statistically significant (unadjusted OR 0.34, 95% CI 0.09–1.28; *p* = 0.173).

### 3.4. Surgical Management and Macroscopic Intraoperative Findings

In the postnatal cohort, operative approach was documented in 45/48 patients (93.8%), with laparoscopy performed in 35/45 (77.8%). Detorsion was documented or confirmed in 38/48 patients (79.2%). Complete oophorectomy or adnexectomy was performed in 13/48 patients (27.1%), whereas ovarian tissue was preserved in the remaining 35 patients (72.9%). Detailed operative procedures are presented in Supplementary Table S3.

Among the 13 postnatal patients undergoing complete oophorectomy or adnexectomy, operative findings consistently indicated advanced ovarian or adnexal nonviability. No postnatal oophorectomy was documented as being performed primarily because of uncontrolled bleeding or suspected malignancy.

In the overall cohort (*n* = 56), macroscopic ovarian appearance after detorsion was classified as complete, partial, or absent color change. Complete color change was observed in 19 patients (33.9%), partial change in 13 (23.2%), and absent change in 24 (42.9%). All ovaries demonstrating complete or partial color change were preserved. Among the 24 ovaries without visible color improvement, 21 (87.5%) underwent oophorectomy, whereas three (12.5%) were preserved despite the absence of macroscopic improvement (Table 3).

Oophoropexy was performed in eight postnatal patients following detorsion of the affected ovary; prophylactic fixation of the contralateral ovary was not performed.

Documented recurrent ovarian torsion occurred in three patients (5.4%) at 3, 5, and 36 months after the index operation. Two were ipsilateral retorsions of previously preserved ovaries and one was contralateral torsion following prior adnexectomy. All required repeat surgery; ovarian preservation was achieved in two cases and adnexectomy in one. Detailed case-level characteristics are provided in Supplementary Table S4.

Given the small number of recurrence events and non-random use of oophoropexy, these findings were considered descriptive.

### 3.5. Temporal Trends in Clinical Management

In the postnatal cohort, use of laparoscopy increased from 33.3% (2/6) in 2010–2017 to 84.6% (33/39) in 2018–2025 (*p* = 0.016), while documented preoperative ultrasonography increased from 62.5% (5/8) to 92.5% (37/40; *p* = 0.050). Gynecologist consultation was numerically more frequent and oophorectomy numerically less frequent in the later period, although neither difference was statistically significant. Oophoropexy rates were similar between periods. These temporal comparisons were exploratory.

### 3.6. Associated Pathology

Associated pathology was identified in 37/56 patients (66.1%), most commonly simple ovarian cysts (22/37, 59.5%) and hemorrhagic ovarian cysts (7/37, 18.9%). Detailed associated pathological findings are provided in Supplementary Table S5. The degree of torsion did not differ significantly between patients with and without associated pathology (median 720° [IQR 360–720] vs. 720° [IQR 360–900]; *p* = 0.494).

### 3.7. Comparison of Ovarian Outcomes in the Postnatal Cohort

In the postnatal cohort, 13/48 patients (27.1%) underwent complete oophorectomy or adnexectomy, whereas ovarian tissue was preserved in 35/48 (72.9%). No statistically significant differences were observed between the groups in age, symptom duration, admission-to-surgery interval, degree of torsion, preoperative gynecologist consultation, documented preoperative ultrasonography, or associated pathology (Table 4).

In a sensitivity analysis excluding symptom-duration values ≥168 h, symptom duration remained not significantly different between the oophorectomy and ovarian-preservation groups (median 24 h [IQR 10–72] vs. 12 h [IQR 8–66]; *p* = 0.344).

### 3.8. Exploratory Multivariable Analysis of Factors Associated with Oophorectomy

In the postnatal cohort, exploratory multivariable logistic regression including age, preoperative gynecologist consultation, and calendar period identified no independent associations with oophorectomy (Table 5). Preoperative gynecologist consultation was associated with a lower point estimate for the odds of oophorectomy, but the confidence interval was wide and included the null value (adjusted OR 0.41, 95% CI 0.09–1.78; *p* = 0.234). Firth penalized logistic regression yielded similar results. Given the limited number of oophorectomy events (*n* = 13), these findings should be interpreted as exploratory.

### 3.9. Follow-Up and Postoperative Complications

Documented postoperative outpatient follow-up was available for 51/56 patients (91.1%), with a median institutional follow-up of 3 months (IQR, 2–8 months). In the postnatal cohort, follow-up duration did not differ significantly between patients undergoing complete oophorectomy/adnexectomy and those with ovarian preservation (median 3 [IQR, 2–8] vs. 3 [IQR, 2–6.75] months; *p* = 0.682). These data represent documented institutional follow-up rather than complete longitudinal surveillance.

Postoperative complications were documented in two patients (3.6%), comprising one Clavien–Dindo grade II and one grade I event. No postoperative infections, thromboembolic events, or deaths were recorded during documented follow-up. Recurrence-related procedures were analyzed separately.

## 4. Discussion

This 15-year single-center study highlights several clinically relevant aspects of pediatric ovarian torsion. Ovarian preservation was achieved in 62.5% of patients, while prenatal torsion represented a clinically distinct subgroup with a substantially higher likelihood of oophorectomy. In the postnatal cohort, preoperative gynecologist consultation was associated with a numerically lower oophorectomy rate, although this association was not statistically significant in either unadjusted or adjusted analyses. Overall, these findings support distinguishing prenatal from postnatal torsion and favor ovarian preservation whenever technically feasible and oncologically appropriate.

The overall oophorectomy rate was strongly influenced by the eight patients with torsion retrospectively classified as prenatal, all of whom underwent oophorectomy. Prenatal torsion differs from acute postnatal torsion because the timing of the event is often uncertain and irreversible ischemic changes may already be present at diagnosis; similarly, Kurtmen et al. reported necrosis and dystrophic calcification in all prenatal cases in their series [7]. Interpretation of overall surgical outcomes should also consider the 15-year study period, during which ovarian-sparing practice, operative approach, and specialist involvement evolved. Previous studies have likewise reported temporal and age-related variation in surgical management [4,5], as well as greater use of ovary-sparing surgery with pediatric gynecologic involvement [6,8].

In the postnatal cohort, preoperative gynecologist consultation was associated with a numerically lower oophorectomy rate, but this association was not statistically significant in either unadjusted or adjusted analyses. Previous studies have similarly suggested that specialist gynecologic involvement may be associated with a greater use of ovary-sparing surgery [9]. However, consultation was not randomly assigned and may have been influenced by patient age, case complexity, surgeon preference, specialist availability, and temporal changes in practice. Accordingly, our findings should not be interpreted as evidence that gynecologist consultation itself improves ovarian preservation.

Timely diagnosis remains important because prolonged torsion may lead to progressive vascular compromise and tissue necrosis [10,11,12]. In our postnatal cohort, the admission-to-surgery interval did not differ significantly according to ovarian outcome; however, this measure captures only the in-hospital component of the diagnostic pathway and not prehospital delay or total ischemic duration. Ultrasonographic findings were heterogeneous, consistent with previous evidence that preserved Doppler flow does not exclude torsion [13,14,15]. Imaging should therefore be interpreted within the overall clinical context and should not delay operative evaluation when torsion is strongly suspected [14,16].

Associated pathology was common, most frequently simple ovarian cysts, but neither associated pathology nor the degree of torsion was significantly associated with ovarian outcome in the postnatal cohort. These findings argue against using torsion degree in isolation to determine ovarian viability. When a solid or complex mass raises concern for malignancy, operative decision-making is necessarily more nuanced; however, tumor markers and other malignancy-risk features were not systematically available in our cohort. Current ovarian-preserving principles favor preservation of residual ovarian tissue when malignancy is considered unlikely [16].

Macroscopic color improvement after detorsion was associated with ovarian preservation in our cohort; however, intraoperative appearance represents a subjective visual assessment and should not be equated with objective reperfusion or subsequent ovarian function. Importantly, three ovaries without visible color improvement were nevertheless preserved. Previous studies evaluating postoperative ovarian morphology, Doppler perfusion, follicular activity, and ovarian reserve similarly emphasize that intraoperative appearance does not reliably determine subsequent functional recovery [16,17,18].

Recurrent torsion was uncommon, occurring in three patients, consistent with the relatively low recurrence rates reported in pediatric series [19]. Although oophoropexy may be considered in selected patients at increased risk of recurrence, its routine role remains uncertain [20]. Given the small number of oophoropexies and recurrences and the non-random selection of patients for fixation, our findings cannot establish its effectiveness. Postoperative morbidity was also low, with only two documented complications, precluding meaningful comparative analysis.

This study has several limitations. Its retrospective, single-center design and relatively small sample size limit statistical power and generalizability, particularly for uncommon outcomes. Secondary and post hoc analyses were exploratory, and multiple comparisons increase the possibility of type I error. Clinical practice evolved over the 15-year study period, and the limited number of oophorectomy events restricted multivariable adjustment, leaving potential for residual confounding. Several measures were dependent on retrospective documentation: exact diagnostic and treatment timestamps were incomplete, ultrasonographic reporting was not standardized, and macroscopic color change reflected subjective surgeon assessment. The threshold for oophorectomy was also not standardized. Importantly, ovarian preservation at surgery cannot be equated with long-term ovarian viability or function because standardized longitudinal data on ovarian morphology, follicular activity, endocrine function, menstrual outcomes, and fertility were unavailable. Follow-up was limited to institutional records, and gynecologist consultation and oophoropexy were not randomly assigned, introducing potential confounding by indication. Finally, prenatal torsion substantially influenced overall outcomes and was therefore analyzed separately from acute postnatal torsion where clinically appropriate.

## 5. Conclusions

Ovarian preservation should remain a primary objective in pediatric ovarian torsion whenever technically feasible and oncologically appropriate. Prenatal torsion represents a clinically distinct subgroup with a substantially higher likelihood of oophorectomy and should be considered separately from acute postnatal torsion. In the postnatal cohort, preoperative gynecologist consultation was associated with a numerically lower oophorectomy rate, although this association was not statistically significant and should not be interpreted as causal. Intraoperative macroscopic appearance should be interpreted cautiously and should not alone determine ovarian viability or predict subsequent functional recovery. Larger, prospective, multicenter studies with standardized management and long-term assessment of ovarian function are needed.

## Figures and Tables

**Table 1:** Baseline Clinical, Diagnostic, and Operative Characteristics of the Study Population (*n* = 56).

Characteristic	Value
Age at diagnosis, years	
Mean ± SD	8.6 ± 5.8
Median (range)	10 (0–17)
Age group, *n* (%)	
Preadolescent (0–12 years)	39 (69.6)
Adolescent (13–18 years)	17 (30.4)
Ovarian torsion retrospectively classified as having occurred prenatally, *n* (%)	8 (14.3)
Calendar period, *n* (%)	
2010–2017	11 (19.6)
2018–2025	45 (80.4)
Duration of symptoms before admission, h, median (IQR) *	18 (8–72)
Time from admission to surgery, h, median (IQR) †	20.3 (4.5–28.7)
Degree of torsion, °, mean ± SD	666.4 ± 326.8
Affected ovary, *n* (%)	
Right	31 (55.4)
Left	25 (44.6)
Preoperative ultrasonography, *n*/N (%) ‡	
Documented	42/48 (87.5)
No documented preoperative ultrasonography	6/48 (12.5)
Preoperative gynecologist consultation, *n*/N (%) ‡	31/48 (64.6)
Associated pathology, *n* (%)	37 (66.1)
Operative approach, *n*/N (%) §	
Laparoscopy	38/53 (71.7)
Laparotomy	10/53 (18.9)
Conversion from laparoscopy to laparotomy	5/53 (9.4)
Primary ovarian outcome, *n* (%)	
Ovarian preservation	35 (62.5)
Complete oophorectomy/adnexectomy	21 (37.5)
Documented recurrent torsion, *n* (%)	3 (5.4)
Postoperative complications, *n* (%)	2 (3.6)
Institutional follow-up duration, months, median (IQR; range)	3 (2–8; 7 days–24 months)
Documented postoperative outpatient follow-up, *n* (%)	51 (91.1)

* Symptom-duration analyses excluded prenatal cases and patients in whom the onset of the acute symptomatic episode could not be reliably determined. † Admission-to-surgery interval was available for 45/48 postnatal patients. ‡ Preoperative ultrasonography and gynecologist-consultation data are presented for the postnatal cohort (*n* = 48) because prenatal torsion represents a clinically distinct diagnostic pathway. § Operative approach was documented in 53/56 patients. Percentages were calculated using patients with available operative-approach data. Follow-up duration was calculated among the 51 patients with at least one documented postoperative outpatient visit.

**Table 2:** Characteristics of Patients With and Without Preoperative Gynecologist Consultation in the Postnatal Cohort (*n* = 48).

Characteristic	Gynecologist Consultation (*n* = 31)	No Gynecologist Consultation (*n* = 17)	*p*-Value
Age, years, median (IQR)	13.0 (9.5–14.5)	7.0 (2.0–12.0)	0.004
Symptom duration, h, median (IQR) *	24 (8–84)	12 (9–48)	0.556
Degree of torsion, °, median (IQR)	720 (360–1080)	720 (360–720)	0.127
Associated pathology, *n* (%)	19 (61.3)	9 (52.9)	0.760
Suspected ovarian/adnexal mass on preoperative ultrasonography, *n* (%)	17 (54.8)	4 (23.5)	0.067
Calendar period 2018–2025, *n* (%)	28 (90.3)	12 (70.6)	0.112
Laparoscopic approach, *n*/N (%) †	24/29 (82.8)	11/16 (68.8)	0.455
Complete oophorectomy/adnexectomy, *n* (%)	6 (19.4)	7 (41.2)	0.173

* Symptom duration was unavailable in one patient in the non-consultation group. † Operative approach was documented in 29/31 patients with consultation and 16/17 patients without consultation.

**Table 3:** Macroscopic Color Change After Detorsion According to Operative Management.

Macroscopic Color Change After Detorsion	Ovarian Preservation, *n* (%)	Oophorectomy, *n* (%)	Total, *n*
Complete	19 (100%)	0 (0%)	19
Partial	13 (100%)	0 (0%)	13
Absent	3 (12.5%)	21 (87.5%)	24
Total	35 (62.5%)	21 (37.5%)	56

**Table 4:** Comparison of Clinical and Diagnostic Characteristics According to Ovarian Outcome in the Postnatal Cohort (*n* = 48).

Variable	Complete Oophorectomy/Adnexectomy (*n* = 13)	Ovarian Preservation (*n* = 35)	*p*-Value
Age, years, median (IQR)	11.0 (6.0–13.0)	12.0 (7.0–14.0)	0.376
Adolescent age group, *n* (%)	4 (30.8)	13 (37.1)	0.747
Symptom duration, h, median (IQR) *	36 (11–84)	12 (8–72)	0.253
Admission-to-surgery interval, h, median (IQR) †	5.3 (4.2–28.7)	20.8 (5.6–28.3)	0.460
Degree of torsion, °, median (IQR)	360 (360–720)	720 (360–1080)	0.101
Preoperative gynecologist consultation, *n* (%)	6 (46.2)	25 (71.4)	0.173
Documented preoperative ultrasonography, *n* (%)	10 (76.9)	32 (91.4)	0.323
Associated pathology, *n* (%)	5 (38.5)	24 (68.6)	0.096
Vomiting, *n* (%)	7 (53.8)	17 (48.6)	1.000
Fever, *n* (%)	2 (15.4)	4 (11.4)	0.656
Leukocyte count, ×10^9^/L, median (IQR)	12.1 (10.4–15.73), *n* = 10	11.5 (8.08–14.64), *n* = 32	0.152
CRP, mg/L, median (IQR)	36.3 (3.26–71.44), *n* = 8	0.76 (0.45–3.46), *n* = 19	0.056
Institutional follow-up, months, median (IQR) ‡	3 (2–8)	3 (2–6.75)	0.682

* Symptom duration was available for 47/48 postnatal patients: 12/13 in the complete-oophorectomy group and 35/35 in the ovarian-preservation group. In one patient with a prolonged history of intermittent abdominal pain, onset of the acute symptomatic episode could not be reliably determined, and the value was treated as missing. † Admission-to-surgery interval was available for 45/48 patients: 13/13 in the complete-oophorectomy group and 32/35 in the ovarian-preservation group. Continuous variables were compared using the Mann–Whitney U test and categorical variables using Fisher’s exact test or an appropriate exact test. Laboratory analyses were performed using available cases only; no imputation was performed. ‡ Follow-up duration was calculated among postnatal patients with at least one documented postoperative outpatient visit (12/13 in the complete-oophorectomy/adnexectomy group and 34/35 in the ovarian-preservation group).

**Table 5:** Exploratory Multivariable Logistic Regression Analysis of Factors Associated with Oophorectomy in the Postnatal Cohort (*n* = 48).

Predictor	Conventional aOR (95% CI)	*p*-Value	Firth Penalized OR (95% CI)	*p*-Value
Age, per 1-year increase	0.97 (0.84–1.12)	0.668	0.97 (0.84–1.11)	0.662
Preoperative gynecologist consultation	0.41 (0.09–1.78)	0.234	0.44 (0.11–1.74)	0.240
Calendar period, 2018–2025 vs. 2010–2017	0.83 (0.15–4.70)	0.834	0.81 (0.16–4.03)	0.794

Oophorectomy was the dependent variable. Analyses were restricted to the postnatal cohort (*n* = 48). Calendar period was entered as a binary variable (2010–2017 vs. 2018–2025). Firth penalized logistic regression was performed as a robustness analysis because of the limited number of oophorectomy events (*n* = 13).

## Data Availability

The data that support the findings of this study are available from the corresponding author upon reasonable request. The data are not publicly available due to privacy and ethical restrictions related to pediatric patient information.

## Supplementary Materials

The following supporting information can be downloaded at: https://www.mdpi.com/article/10.3390/children13081118/s1, Table S1: Case-Level Characteristics of Patients with Ovarian Torsion Retrospectively Classified as Prenatal (*n* = 8); Table S2: Preoperative Ultrasonography Findings in the Postnatal Ovarian Torsion Cohort (*n* = 48); Table S3: Operative Approach and Procedures in the Postnatal Ovarian Torsion Cohort (*n* = 48); Table S4: Case-Level Characteristics of Patients with Recurrent Ovarian Torsion (*n* = 3); Table S5: Types of Associated Pathology Identified in Patients with Ovarian Torsion (*n* = 56).
