# Supplementary material for: Clinical Characteristics and Surgical Outcomes of Pediatric Ovarian Torsion: A 15-Year Single-Center Experience
Source: Children (Basel). 2026 Aug 21;13(8):1118. doi: 10.3390/children13081118 (PMC13510985; doi:10.3390/children13081118)
Supplement: Supplementary file 1 [file children-13-01118-s001.zip › children-4492767-supplementary.pdf]

Supplementary Table S1. Case-Level Characteristics of Patients with Ovarian Torsion Retrospectively Classified as Prenatal (n = 8).

Case	Age at surgery (days)	Diagnostic information / postnatal symptoms	Preoperative imaging	Side / torsion	Operative findings	Surgical procedure	Histopathology
1	12	Prenatal classification; external imaging mentioned; no acute postnatal symptoms documented	External US; detailed report unavailable	Right / 720°	Advanced necrobiotic changes of the adnexa	Adnexectomy	Not available
2	7	Retrospectively classified as prenatal; no acute postnatal symptoms documented	Ovarian cyst with dense internal content	Left / 360°	Gangrenous ovary and fallopian tube	Adnexectomy	Not available
3	14	Retrospectively classified as prenatal; evaluated after birth; no acute postnatal symptoms documented	Complex cystic adnexal lesion	Right / 360°	Gangrenous ovary and fallopian tube	Adnexectomy	Not available
4	11	Retrospectively classified as prenatal; evaluated after birth; no acute postnatal symptoms documented	Complex cystic lesion; absent Doppler flow	Right / 360°	Gangrenous ovary and fallopian tube	Adnexectomy	Not available
5	6	Retrospectively classified as prenatal; no acute postnatal symptoms documented	Previously documented ovarian cyst	Left / 360°	Avital ovary	Oophorectomy	Not available
6	8	Retrospectively classified as prenatal; evaluated after birth; no acute postnatal symptoms documented	Complex cystic abdominal/adnexal lesion; absent Doppler flow	Left / 360°	Gangrenous ovary and fallopian tube	Adnexectomy	Not available
7	9	Retrospectively classified as prenatal; no acute postnatal symptoms documented	Detailed institutional US description unavailable	Left / 360°	Autoamputation of ovary and fallopian tube	Adnexectomy	Not available

Case	Age at surgery (days)	Diagnostic information / postnatal symptoms	Preoperative imaging	Side / torsion	Operative findings	Surgical procedure	Histopathology
8	10	Intrauterine ovarian cyst documented; no acute postnatal symptoms documented	Large cystic lesion	Left / 360°	Autoamputation	Cyst puncture and oophorectomy	Not available

Supplementary Table S2. Preoperative Ultrasonography Findings in the Postnatal Ovarian Torsion Cohort (n = 48).

Ultrasonographic finding	n/N (%)
Documented preoperative ultrasonography	42/48 (87.5)
No documented preoperative ultrasonography	6/48 (12.5)
**Findings among patients with documented ultrasonography (n = 42)**	
Ovarian enlargement	8/42 (19.0)
Peripheral follicles/cysts specifically described	2/42 (4.8)
Stromal edema specifically described	0/42 (0)
Free pelvic/periovarian fluid	10/42 (23.8)
Whirlpool sign specifically described	0/42 (0)
Suspected ovarian/adnexal mass or prominent cystic/mass-like lesion	21/42 (50.0)
Doppler assessment specifically documented	23/42 (54.8)
— No detectable flow	13/23 (56.5)
— Reduced/partial or peripheral-only flow	3/23 (13.0)
— Preserved ovarian flow	2/23 (8.7)
— Avascular lesion with preserved ovarian flow	2/23 (8.7)

Ultrasonographic finding	n/N (%)
— Technically unreliable/not assessable	3/23 (13.0)

Findings not explicitly described in the original written ultrasonography report were classified as "not reported" rather than assumed to be absent. Doppler categories refer to the dominant documented Doppler finding. "Suspected ovarian/adnexal mass or prominent cystic/mass-like lesion" included clearly described cystic, solid, complex, or mass-like adnexal/ovarian formations on preoperative ultrasonography.

Supplementary Table S3. Operative Approach and Procedures in the Postnatal Ovarian Torsion Cohort (n = 48).

Operative characteristic	n
Operative approach*	
Laparoscopy	35
Laparotomy	6
Conversion from laparoscopy to laparotomy	4
Operative procedures†	
Detorsion‡	38
Excision/resection of ovarian or paratubal lesion	11
Fenestration/unroofing of cystic lesion	5
Cyst/tubal puncture	3
Oophoropexy	8
Complete oophorectomy/adnexectomy	13
Partial ovarian resection with ovarian preservation	1
Salpingectomy with ovarian preservation	1
Appendectomy	13

Operative characteristic	n
Hernia repair	4

Operative approach was documented in 45 of 48 postnatal patients;
†Operative procedures were not mutually exclusive; more than one procedure could be performed in the same patient.
‡Detorsion was considered documented when explicitly recorded in the operative data or otherwise confirmed from the available operative record. Absence of explicit documentation of detorsion was not interpreted as evidence that detorsion had not been attempted.

Supplementary Table S4. Case-Level Characteristics of Patients with Recurrent Ovarian Torsion (n = 3).

Case	Age at Index Surgery	Index Side / Torsion	Associated Pathology	Initial Surgical Management	Oophoropexy at Index Surgery	Recurrence	Management of Recurrence
1	6 years	Left / 360°	None documented	Adnexectomy	No	Contralateral right ovarian torsion, 36 months later	Detorsion with ovarian preservation; no fixation
2	16 years	Right / 360°	None documented	Detorsion with ovarian preservation	No	Ipsilateral right ovarian retorsion, 3 months later	Adnexectomy
3	6 years	Left / 1080°	Ovarian cyst	Detorsion with ovarian preservation	Yes; fixation to the lateral pelvic wall with one nonabsorbable suture	Ipsilateral left ovarian retorsion, 5 months later; adhesive band identified as the axis of torsion	Detorsion with ovarian preservation; no repeat fixation

Supplementary Table S5. Types of Associated Pathology Identified in Patients with Ovarian Torsion (n = 56).

Associated pathology	n	% of associated pathology (n = 37)	% of total cohort (n = 56)
Simple ovarian cyst	22	59.5	39.3
Hemorrhagic ovarian cyst	7	18.9	12.5
Paratubal cyst	3	8.1	5.4
Inguinal hernia	2	5.4	3.6
Dermoid cyst	1	2.7	1.8
Teratoma	1	2.7	1.8
Hematosalpinx	1	2.7	1.8
Total	37	100.0	66.1
